# Structural Insights into *Pseudomonas aeruginosa* Exotoxin A–Elongation Factor 2 Interactions: A Molecular Dynamics Study

**DOI:** 10.1021/acs.jcim.3c00064

**Published:** 2023-02-20

**Authors:** Asma Gholami, Dariush Minai-Tehrani, Sayyed Jalil Mahdizadeh, Patricia Saenz-Mendez, Leif A. Eriksson

**Affiliations:** † Department of Chemistry and Molecular Biology, University of Gothenburg, Göteborg 405 30, Sweden; ‡ Faculty of Life Sciences and Biotechnology, Shahid Beheshti University, Tehran 1983969411, Iran; § Department of Engineering and Chemical Sciences, Karlstad University, 651 88 Karlstad, Sweden

## Abstract

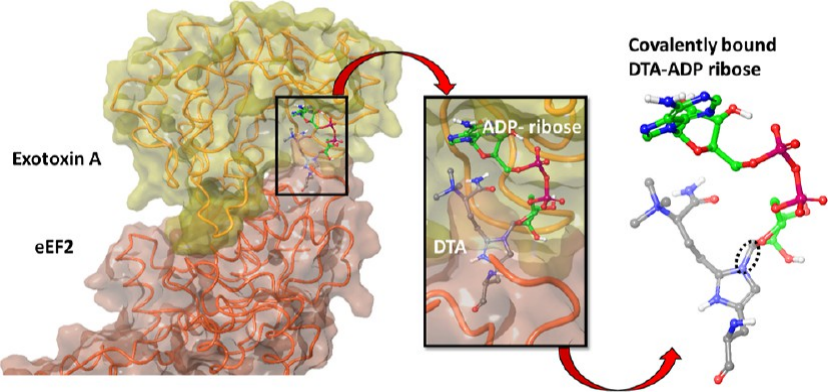

Exotoxin A (ETA) is an extracellular secreted toxin and a single-chain polypeptide with A and B fragments that is produced by *Pseudomonas aeruginosa*. It catalyzes the ADP-ribosylation of a post-translationally modified histidine (diphthamide) on eukaryotic elongation factor 2 (eEF2), which results in the inactivation of the latter and the inhibition of protein biosynthesis. Studies show that the imidazole ring of diphthamide plays an important role in the ADP-ribosylation catalyzed by the toxin. In this work, we employ different *in silico* molecular dynamics (MD) simulation approaches to understand the role of diphthamide versus unmodified histidine in eEF2 on the interaction with ETA. Crystal structures of the eEF2–ETA complexes with three different ligands NAD^+^, ADP-ribose, and βTAD were selected and compared in the diphthamide and histidine containing systems. The study shows that NAD^+^ bound to ETA remains very stable in comparison with other ligands, enabling the transfer of ADP-ribose to the N3 atom of the diphthamide imidazole ring in eEF2 during ribosylation. We also show that unmodified histidine in eEF2 has a negative impact on ETA binding and is not a suitable target for the attachment of ADP-ribose. Analyzing of radius of gyration and COM distances for NAD^+^, βTAD, and ADP-ribose complexes revealed that unmodified His affects the structure and destabilizes the complex with all different ligands throughout the MD simulations.

## 1 Introduction

The eukaryotic elongation factor 2 (eEF2) is an essential GTPase enzyme, playing a key role during protein synthesis. It assists in elongating the developing polypeptide chain by one amino acid at a time.^1^ eEF2 is also unique as it carries a ubiquitous and exclusive post-translational modification of one histidine residue (His699 in yeast and His715 in mammals) into diphthamide: 2-[3-carboxyamido-3-(trimethylammonio)propyl]histidine, DTA. Although the precise role of DTA remains unknown, its absence has been associated with altered translational fidelity,^2−4^ indicating that the diphthamide unit is vital in higher eukaryotic cells.^5^ Moreover, eEF2 is not only essential, but it is also vulnerable to toxins. The name diphthamide refers to it being the target site of the diphtheria toxin (DT, *Corynebacterium diphtheriae*) and other NAD^+^-dependent ADP ribosylating toxins, such as *Vibrio cholera* cholix toxin (CT) and exotoxin A (ETA, *Pseudomonas aeruginosa*).^6^

**Figure 1:**
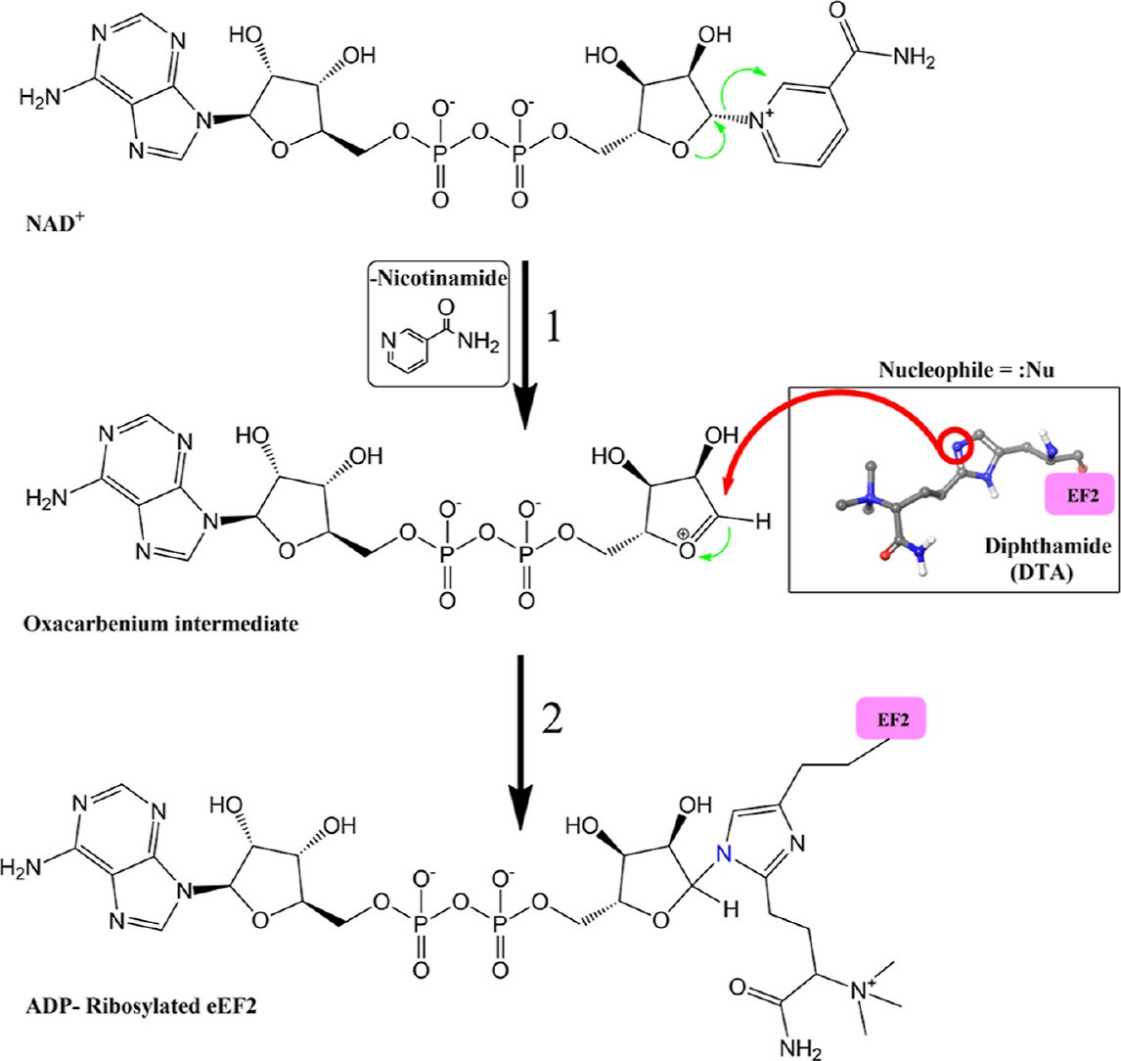
Proposed mechanism of diphthamide modification by ADP-ribosylases (ETA, DT, and CT).

*P. aeruginosa* is a well-known opportunistic pathogen, affecting immunocompromised patients with cystic fibrosis, cancer, AIDS, and burn victims,^7−9^ and is sadly known for its resistance to major classes of antibiotics.^10−12^ In *P. aeruginosa*, the most toxic secreted factor is ETA, which has an LD_50_ of 0.2 μg/kg upon intraperitoneal injection in mice.^13−15^ ETA is an AB toxin of 66 kDa, composed of 613 residues, reported for the first time in 1966.^16^ The modification of diphthamide promoted by ETA involves the transfer of an ADP-ribose moiety from NAD^+^ to the N3 atom of the diphthamide imidazole ring in eEF2, most likely following an S_N_1 mechanism (Figure 1).^17−22^ Thus, the unique and essential DTA in eEF2 is also the site of ADP-ribosylation of lethal toxins,^23^ irreversibly inactivating the translation function of eEF2 and leading to cell death.^13,17,24,25^

**Figure 2:**
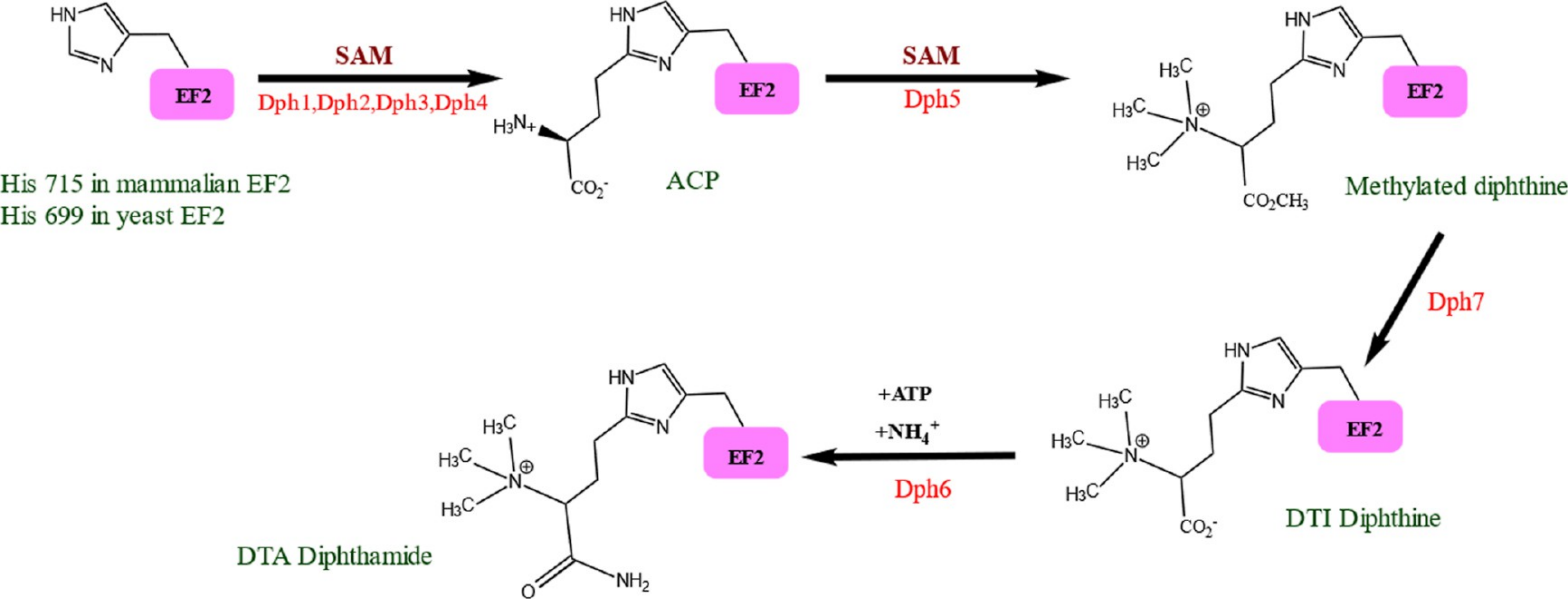
Biosynthetic pathway of diphthamide in eukaryotes (adapted from ref (28)).

In eukaryotic cells, DTA generation from histidine involves the seven enzymes Dph1-Dph7, in four steps (Figure 2).^26,27^

Interestingly, the unmethylated intermediate ACP is not ribosylated by the toxins. In addition, the trimethylated carboxylate precursor DTI, lacking the amide functional group, is ribosylated at a lower rate than DTA.^18,29,30^ These pieces of evidence clearly emphasize the importance of the amide and the quaternary nitrogen for ADP-ribosylation. However, their precise roles when targeted by ETA are as yet not established.

In this work, we have focused on complexes between eEF2 (containing either the post-translational modification DTA or the original precursor His715) and ETA. We have carried out a combination of *in silico* studies including homology modeling and MD simulations, to shed light on the interactions that make eEF2 the target of NAD^+^-dependent ADP-ribosylase toxins and in particular *P. aeruginosa* ETA.

## 2 Methodology

### 2.1 Homology Modeling and Protein Data

Due to the absence of a crystal structure of human eEF2, homology modeling was used to obtain the 3D structure for the *in silico* studies. The FASTA sequence of eEF2 consisting of 858 amino acids (Figure S1) was retrieved from UniProt (KB-P13639) and used as a target for subsequent homology modeling in YASARA v. 20.12.24 (www.yasara.org).^32,33^ The crystal structure of ADP-ribosylated ribosomal translocase from *Saccharomyces cerevisiae* (PDB ID:1U2R) was identified as the main template. All homology modeling was conducted using the default settings (Table S1) in the macro hm_build.mcr and involved BLAST searches, multiple sequence alignments, loop modeling, refinement, and molecular dynamics (MD)-based simulated annealing minimization, leading to the generation of a total of 10 models. Following quality assessment of the generated models, a final hybrid model was generated (default by the homology modeling macro) and subjected to unconstrained minimization. His715 was subsequently modified in the final model by constructing the DTA substituent using the builder function in Molecular Operating Environment 2019.01 (MOE; www.chemcomp.com), followed by additional energy minimization.

Four protein structures of *P. aeruginosa* ETA were downloaded from the Protein Data Bank (www.rcsb.org) and further analyzed: wild type protein (PDB ID: 1IKQ),^34^ the structure of the eEF2 -ETA-NAD^+^ complex (PDB ID: 2ZIT),^35^ ADP-ribosylated eEF2 in complex with ETA (PDB ID: 1ZM2), and the eEF2–ETA complex with the NAD^+^-analogue βTAD bound (PDB ID: 1ZM4).^20^ In all these structures, EF2 is from *S. cerevisiae*.

The sequences were aligned, the structures superposed, and compared.

### 2.2 System Preparation

All downloaded complexes were prepared with the Protein Preparation Wizard in Maestro (Schrödinger 2021-4, www.schrodinger.com). In the preprocessing stage, hydrogen atoms and disulfide bonds were added to the initial coordinates; then, bond orders and completion of missing loops and side chains were selected in order to correct defects, using Prime.^42^ All water molecules located beyond 5.0 Å from the ligands were deleted from the systems. To find the most likely protonation states of the side chains and the energy penalties associated with alternate protonation states, pH = 7 ± 2.0 was used. The protein hydrogen bond assignment was then optimized in the H-bond Refine Tab using sample water orientations and PROPKA^43^ pH = 7. For restrained minimizations, the root-mean-square deviation (rmsd) for heavy atom convergence was set to 0.3 Å. The OPLS4 force field^36^ was employed, and hydrogen atoms were minimized while allowing sufficient heavy atom movement to relax strained bonds, angles, and clashes.

### 2.3 Prime MM-GBSA

The ETA–eEF2 complexes were refined using the Prime MM-GBSA engine in Maestro, with the VSGB solvation model.^44^ Residues within 5 Å from the ligand were considered flexible, and OPLS4 was selected as the force field.^36^

### 2.4 Molecular Dynamics Simulations

All MD simulations were performed in triplicate for 200 ns using NPT ensembles and the OPLS4 force field,^36^ with the Desmond MD engine^37^ implemented in Schrödinger (Schrödinger 2021-4, www.schrodinger.com). The TIP3P force field was used to model water molecules.^38^ Periodic boundary conditions were applied with a 10 Å water buffer around the protein in a cubicoid simulation box. The appropriate number of counterions (i.e., Na^+^/Cl^–^) were added to neutralize the system and to obtain a physiological salt concentration of 150 mM. The temperature 300 K and pressure 1 atm were controlled using the Nose–Hoover thermostat (1 ps relaxation time) and the Martyna–Tobias–Klein barostat (isotropic coupling), respectively.^39,40^ Electrostatic forces were treated using particle-mesh Ewald summation^45^ with a cut-off of 9 Å both for electrostatics and van der Waals interactions. The “default” relaxation protocol consisted of several steps as follows: (1) NVT Brownian Dynamics with restraints on solute heavy atoms at *T* = 10 K for 100 ps, (2) NVT MD simulation at *T* = 10 K with restraints on solute heavy atoms for 12 ps, (3) NPT MD simulation at *T* = 10 K with restraints on solute heavy atoms for 12 ps, (4) NPT MD simulation at *T* = 300 K with restraints on solute heavy atoms for 12 ps, and (5) NPT MD simulation at *T* = 300 K without restraints for 24 ps. This protocol was followed by 200 ns MD production simulations in triplicate. The total simulation length adding up all systems in the current study amounts to 6.6 μs.

### 2.5 Clustering

From the MD trajectories, clustering of the resulting structures was performed to group similar molecular conformations into distinct sets such that the structures in each cluster are more similar to each other compared to structures in any other cluster. This gives a refined view of how a given molecule is sampling the conformational space and allows direct characterization of the separate conformational sub-states visited during the MD simulation.

To this end, the Desmond Trajectory clustering tool in Maestro (Schrödinger 2021-4, www.schrodinger.com) was employed, whereby the backbone atoms for each protein were introduced in the Atom Specification Language (ASL) dialogue box, setting the initial number of clusters to 3. After running the clustering tool, the structures of each complex with the largest number of similar conformations were selected for the next step; that is, identifying the complex structures with the lowest energies and highest stabilities.

### 2.6 Binding Pose Metadynamics

The main principle in binding pose metadynamics (BPMD) is that under the same biasing force, ligands that are not stably bound to the receptor will experience more fluctuations or jumps in their rmsd in comparison with the stably bound ones. During the course of the metadynamics simulations, the score value is indicating the stability of the ligand in the complex. Before the actual metadynamics run, MD clustering as outlined above was performed to find the most representative conformation and remove any bad contacts and/or strain in the initial starting structure.

For each system, 10 independent metadynamics simulations of 10 ns each were performed in Maestro (Schrodinger 2021-4, www.schrodinger.com). The rmsd of the ligand heavy atoms relative to their starting positions was used as collective variable.

## 3 Results and Discussion

### 3.1 Homology Modeling and Protein Data

Having constructed the homology model of human eEF2 with either histidine or DTA as residue 715, quality assessment based on estimation of the Z-score using the QMEAN server (swissmodel.expasy.org/qmean) was conducted for both models. Additional validation with Ramachandran plots of backbone dihedrals generated in MOE v. 2019.01 showed that the vast majority of residues were situated in favorable or allowed regions. The final, complete 3D models of human eEF2 were used as the starting points for further simulations and analysis.

### 3.2 Comparison of Proteins

**Figure 3:**
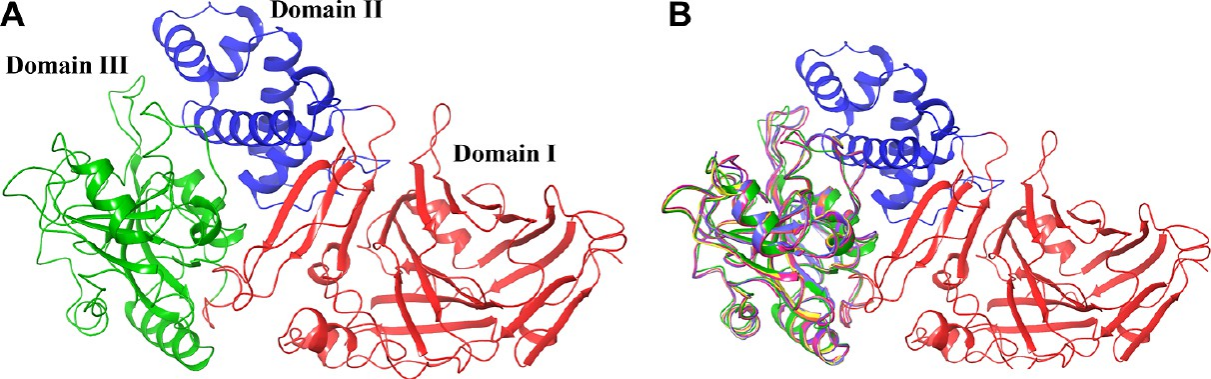
(A) Different domains of ETA (PDB ID: 1IKQ) highlighted: receptor-binding domain I (red), transmembrane-targeting domain II (dark blue), and catalytic domain III (green). (B) Superposition of the 3D structures of domain III of the toxins; 1IKQ in green, 2ZIT in pink, 1ZM2 in yellow, and 1ZM4 in purple.

The PDB structures (1IKQ, 2ZIT, 1ZM2, and 1ZM4) were selected based on the lack of mutations in ETA, the presence of a relevant ligand (such as NAD^+^ or β-TAD), and the resolution. Except for 1IKQ, which consists only of *P. aeruginosa* ETA, all other structures correspond to different complexes of *P. aeruginosa* ETA and *S. cerevisiae* eEF2. 1IKQ corresponds to wild-type ETA, with all three structural domains. These are individually responsible for receptor binding (domain I, 1a: 1–252, 1b: 365–404), transmembrane targeting (domain II, 253–364), and ADP-ribosyl transferase (domain III, the enzymatic domain, 405–613), Figure 3A. Domain III is responsible for the ADP ribosyl transferase activity. ETA in 1IKQ corresponds to the extracellular state of the toxin, before entering the cell and undergoing proteolysis.^34^ Thus, 1KIQ was selected as the reference, onto which we superposed domain III from the other selected crystal structures (Figure 3B).

When comparing the different ETA containing complexes with that of the full toxin protein (1IKQ), it becomes clear that all four proteins are structurally very similar. The lowest rmsd compared with the full-length ETA (1IKQ) was obtained for the protein complex of eEF2 with ETA domain III containing NAD^+^ (2ZIT).

### 3.3 Molecular Dynamics Simulations

The aim is to shed light on the role that histidine versus diphthamide play in the interaction between eEF2 and ETA, and on the mechanism of ADP-ribosylation. To this end, we performed and analyzed MD simulations on different systems as follows, in each case with eEF2 containing either His715 or DTA715:

- ETA with either NAD^+^, ADP-ribose, or βTAD as ligand
- eEF2–ETA complex with NAD^+^
- eEF2–ETA complex with ADP-ribose non-bonded
- eEF2–ETA complex with ADP ribose covalently bound to DTA
- eEF2–ETA complex with βTAD

#### 3.3.1 ETA with NAD^+^, ADP-Ribose, or βTAD Ligand

**Figure 4:**
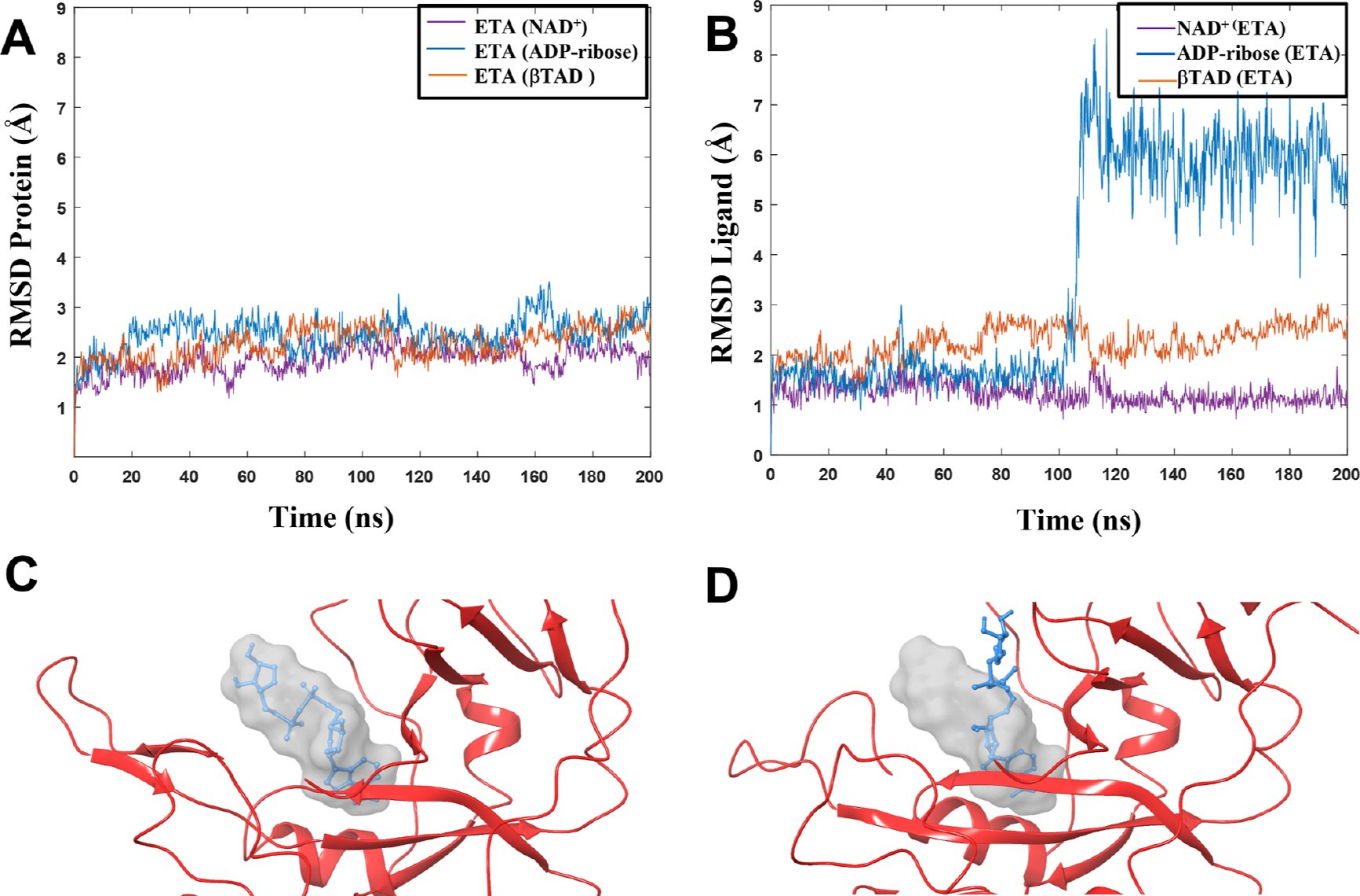
Rmsd values for ETA with three different ligands during one of the 200 ns MD simulation replicas. (A) Rmsd of the proteins. (B) Rmsd of the ligands NAD^+^, βTAD, and ADP-ribose bound to ETA. (C,D) Snapshots of two different frames of the MD trajectory for ETA with ADP-ribose as the ligand. The gray surface shows the cavity of the protein. (C) ADP-ribose (blue color) remains stable in the cavity for the first 100 ns. (D) ADP-ribose (blue color) moving out of the cavity after ∼110 ns.

The first step in the ribosylation of diphthamide involves the release of a nicotinamide moiety from NAD^+^. However, whether the generation of ADP-ribose is taking place in ETA alone or ETA complexed with eEF2 has not been resolved. We thus selected two PDB structures of ETA containing two different ligands. 2ZIT, containing NAD^+^ and 1ZM4, containing βTAD. The ADP-ribose - ETA complex was prepared from 2ZIT by removing the nicotinamide unit from NAD^+^. This leaves the terminal carbon atom positively charged, generating the oxacarbenium intermediate. After protein preparation and minimization, 200 ns MD simulations were conducted in triplicate on all three systems. The lowest rmsd for both protein and ligand are observed when NAD^+^ is bound to ETA (Figure 4A,B). NAD^+^ remains very stable and is not displaced from its binding pocket during the entire trajectory. Analysis of each replica shows very high consistency between the three runs (Table S2 and Figure S4). The NAD^+^ analogue βTAD also remains in the binding site of ETA but with slightly higher average rmsds in each of the replicas (Table S2 and Figure S4).

For the ADP-ribose ETA complex the rmsd value for the protein is essentially the same as for the other two systems in all replicas (Figure 4A and Table S2), whereas the rmsd for the ligand is significantly higher (Figure S4 and Table S2). In replica 3, as shown in Figure 4B, the rmsd of the ligand changes dramatically half way through the MD simulation. After a sudden jump in rmsd for ADP-ribose to 8 Å at *t* = 110 ns, the value remains high during the remainder of the trajectory, indicating that the ligand has moved away from the original binding pose.

As can be seen from Figure 4C, the ligand remains in the cavity of the protein for the first 100 ns. After 100 ns, the ribose carbon atom previously bound to the nicotinamide unit is moving out into the solvent (Figure 4D). In Table S2 and Figure S4, we note that the rmsd of ADP-ribose in replicas 1 and 2 increases to very high values already at the initial stages of the simulations and attains solvent-exposed poses similar to that displayed in Figure 4D. We may thus conclude that ADP-ribose does not remain stable in the binding pocket. The modified interaction between ADP-ribose and ETA indicates that NAD^+^ is most likely to be the complexed ligand in the first step of ETA binding to eEF2, and the release of the nicotinamide unit takes place once the complex between ETA and eEF2 is formed.

**Figure 5:**
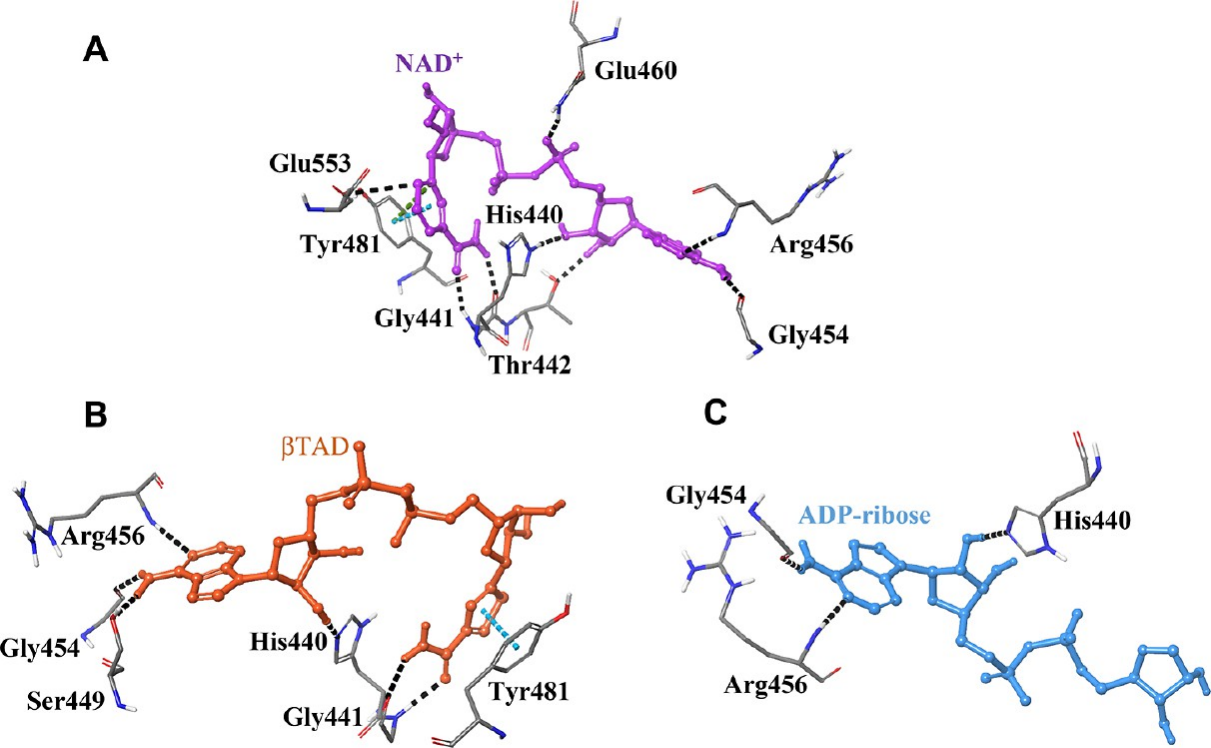
3D views of representative MD snapshots for (A) ETA-NAD^+^, (B) ETA-βTAD, and (C) ETA-ADP-ribose. Different types of interactions are represented in the model by color-dashed lines; blue, black, and green describe π–π stacking, hydrogen bonds, and π-cation interactions, respectively.

To gain further insights on the fact that NAD^+^ and βTAD bound stronger in the active site of ETA than ADP-ribose, we analyzed the interactions in the complexes. Figure 5 shows representative snapshots of the MD trajectory for NAD^+^, βTAD, and ADP-ribose in ETA. As can be seen, several crucial hydrogen bonds are formed during the simulations. The most strictly conserved and vital catalytic residues in ETA are Glu553, His440, and Tyr481.^20^ Glu553 forms a hydrogen bond with the 2′ OH of the ribose moiety of NAD^+^ (Figure 5A). This amino acid with the aid of Tyr481 plays a key role in orienting the dinucleotide substrate for the nucleophilic attack by DTA during the ADP-ribosylation of eEF2.^6,20^ Tyr481 has both π–π stacking and π-cation interactions with the aromatic ring of NAD^+^ but only π–π stacking with βTAD. In addition, Glu460 forms a hydrogen bond with the adenine phosphate of NAD^+^, which is absent in the case of βTAD. Another interaction missing for βTAD is the hydrogen bond with Thr442. These missing interactions are consistent with the lower experimental affinity of βTAD than NAD^+^, validating the docking results. His440 (protonated at Nδ), Gly454, and Arg456 have similar hydrogen bond interactions with the three ligands (Figure 5A–C). When comparing with NAD^+^ and βTAD, the interactions for ADP-ribose are considerably reduced, confirming the higher tendency to dissociate from the active site of the enzyme.

Although both NAD^+^ and βTAD are bound stably to the active site of ETA, it can be inferred based on the protein–ligand interactions, and supported by the rmsd analysis (Figure 4A,B), that NAD^+^ binds stronger than both βTAD and ADP-ribose in the binding pocket of ETA. To further validate these results, BPMD was implemented.

After clustering of the snapshots from the MD simulations, we performed BPMD simulations of NAD^+^, βTAD, and ADP-ribose in the active site of ETA, using Desmond. Pose stability was calculated based on the PoseScore, which is the rmsd of the ligand with respect to the initial ligand heavy atom coordinates. The threshold value for stable ligand binding is a PoseScore ≤ 2 Å. Moreover, to assess the strength of hydrogen bonds between the ligand and protein, the hydrogen bond persistence score (PersScore) was also investigated, using PersScore ≥ 0.6 as the threshold value. For NAD^+^, the calculated values of PoseScore and PersScore were 0.901 and 0.875 Å, respectively, whereas for βTAD, the obtained PoseScore and PersScore were 1.410 and 0.683 Å, respectively. The PoseScore for ADP-ribose was 1.956 Å, and the PersScore was 0.567 Å (Figure S2). These results are consistent with the higher rmsd for the latter, indicating that ADP-ribose is weakly bound to the active site of ETA. The data also indicated that both NAD^+^ and βTAD bind into the active site of ETA. For NAD^+^, the observed rmsd is constant and has a lower value, which indicates higher ligand stability within the active site in comparison with its analogue βTAD (Figure S2). This is further supported by the higher hydrogen-binding PersScore for NAD^+^ and in line with the observations made from the representative MD snapshots (Figure 5A).

#### 3.3.2 eEF2–ETA Complex with NAD^+^

**Figure 6:**
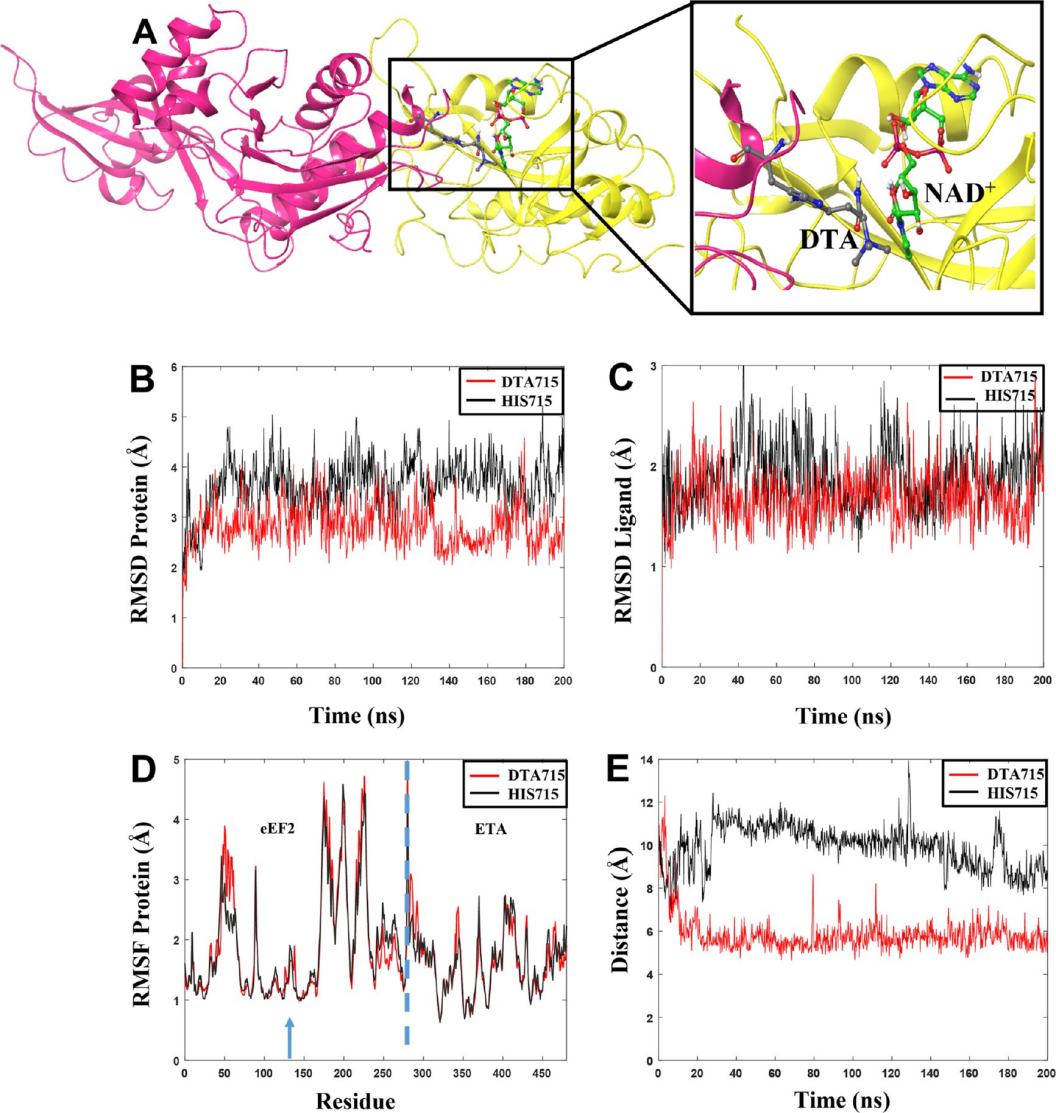
(A) Generated complex between ETA (yellow) containing NAD^+^ and truncated human eEF2 (pink) with DTA in position 715. (B) Rmsd values for proteins in the complex. (C) Rmsd of the ligand NAD^+^ in the presence of DTA (red) or His715 (black) in eEF2. (D) RMSF values for individual residues during the 200 ns MD simulations. The dashed blue line separates the proteins in the complex. Residues 1–279 correspond to eEF2 and residues 280–486 correspond to ETA. The blue arrow shows the location of DTA715 and His715. (E) Fluctuation of the distance between the reactive carbon atom in NAD^+^ and N3 atom in DTA715 (red) or His715 (black).

Next, we analyzed the interactions in the protein complex between eEF2 and ETA, including different ligands to explore the various stages of the reaction. First, the complex with NAD^+^ as ligand was explored to clarify the interactions between the dinucleotide and DTA or the non-modified parent His residue, eventually leading to ribosylation of the former. We overlapped the ETA-NAD^+^–yeast eEF2 complex (PDB ID: 2ZIT) with the homology model of human eEF2 containing the modified residue DTA715. The N-terminal parts located at the other end of eEF2 relative to DTA have different orientations in the yeast and human systems, and thus, residues 1–578 were truncated. This will not affect the interaction with ETA (Figure S3). After superposing the truncated proteins, an rmsd of 1.67 Å was obtained for the segments. After removing the chain corresponding to yeast eEF2, the proteins were merged, and the complex ETA-NAD^+^-*h*EF2 (DTA) was generated (Figure 6A). The complex was prepared in Maestro, minimized, and subjected to MD simulations.

In addition, the complex of ETA-NAD^+^ with human eEF2 containing His instead of DTA in position 715 was generated, prepared in Maestro (using the same protocol as above), minimized, and subjected to MD simulations. The rmsd was calculated for the two complexes studied, that is, for eEF2 having either DTA or His in position 715 (Figure 6B,C, Table S3 and Figure S5).

The calculated rmsds for the protein show that the system containing the unmodified His residue in position 715 has slightly higher fluctuations with larger rmsd values during the MD trajectories (average over the three replicas: 3.65). The observed rmsd for the system with DTA in position 715 is lower (average over three replicas: 3.38 Å), indicating a more stable ETA–eEF2 complex. Moreover, the rmsd for NAD^+^ when eEF2 contains His715 is slightly higher (average: 2.06) than that with DTA715 (average: 1.88), respectively, and displays fluctuations during the entire trajectories (Figures 6C and S5), showing that the complex containing His715 is more prone to structural changes than when DTA is present.

We also analyzed the protein root-mean-square-fluctuations (RMSF), showing those residues fluctuating the most during the MD simulation trajectory (Figure 6D, Table S3, and Figure S5). The analysis of the RMSF, as shown in detail for one of the replicas in Figure 6D, reveals that the flexibility of the unmodified His715 (RMSF: 1.50 Å) is slightly larger than that for DTA715 (RMSF: 1.35 Å; position 715 at the blue arrow) during the trajectory. The same trend is observed in all replicas (Figure S5). The largest overall fluctuations are, consistent with this, seen in all replicas for the complex containing His715 (Table S3).

In order to explore the interaction between NAD^+^ and DTA/His, the distance between the “reactive” ribose carbon atom of NAD^+^ and the N3 atom of the DTA715/His715 imidazole ring in eEF2 was analyzed in one of the replicas (Figure 6E). These two atoms should be sufficiently close to ensure the ribosylation reaction through nucleophilic substitution.

The distance plot for the system containing DTA715 shows that the system rapidly reaches a proper distance (6 Å) between the atoms that may enable the reaction to occur. For the system containing His715, several large conformational changes are observed, and the system does not seem to reach equilibrium. These results show that when His is present in position 715, the atoms that will form the bond are not at a proper distance during the trajectory, having an average value of ∼10 Å.

**Figure 7:**
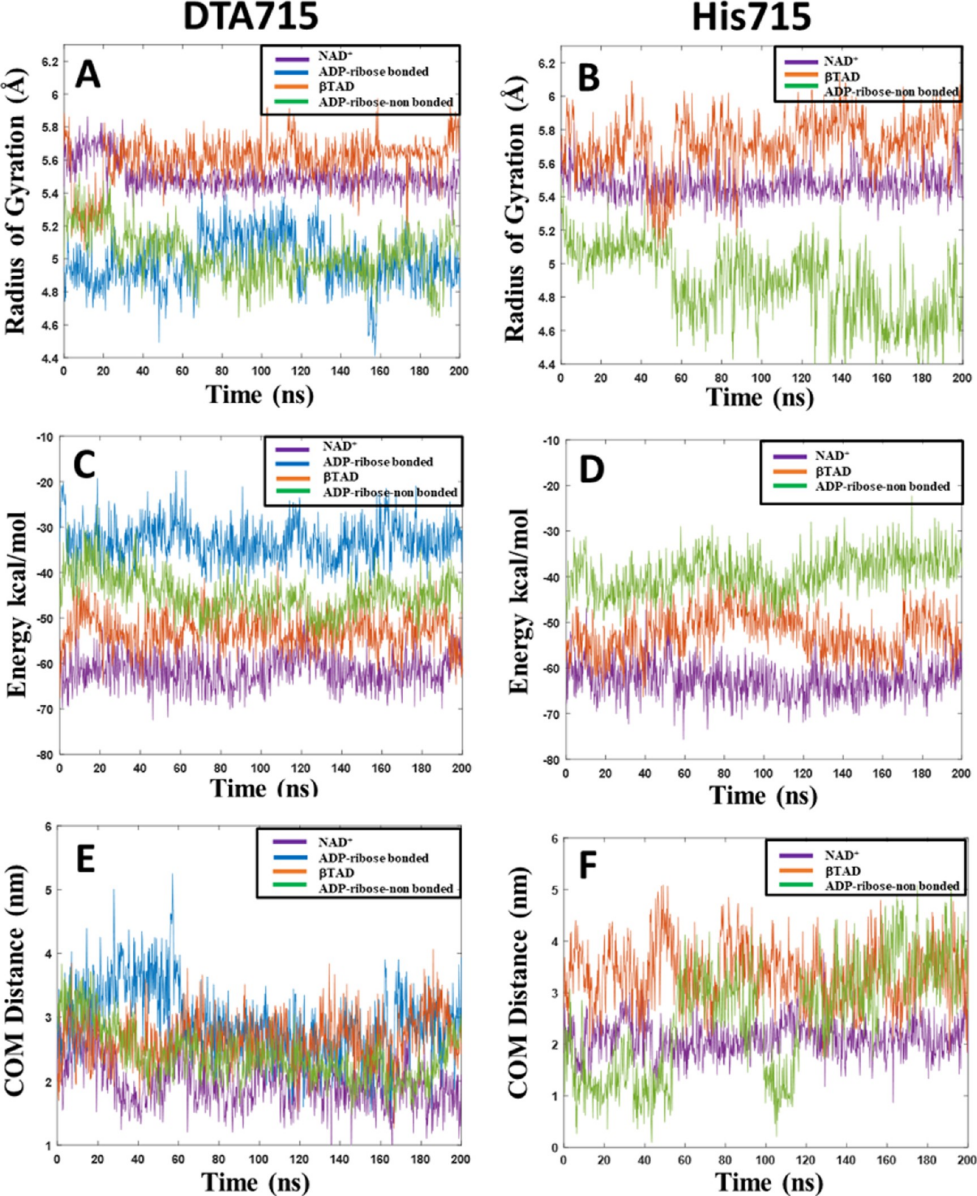
(A,B) Radius of gyration, (C,D) protein–protein interaction energy, and (E,F) distance between the centers of mass (COM) of the proteins for the ETA–eEF2 complexes with DTA in position 715 (left column), and His in position 715 (right column), respectively. Results from one of the three 200 ns MD simulation replicas are shown in each case. Figure legends indicate which ligand that is present in each complex.

To gain deeper insights into the stability of the complexes, additional assessments, such as radius of gyration (*R*_g_), protein–protein interaction energy, and the distance between the centers of mass (COM) of the proteins, were calculated and analyzed for one of the replicas (Figure 7).

The radius of gyration (*R*_g_) remains largely unchanged during the simulations in both complexes containing NAD^+^ as the ligand, with values ca. 5.5 Å (Figure 7A,B).

We also monitored the evolution of the total interaction energies between ETA and eEF2 in both systems (Figure 7C,D). The interaction energies remain stable in both systems throughout the simulations (purple curves), the average interaction energies are highly similar (∼65 kcal/mol) and the largest of all systems studied.

To further explore the dynamic features of the two systems, the change in COM distance between the two proteins was determined. The average values for the two proteins containing DTA715 and His715 with NAD^+^ as the ligand are 1.98 and 2.10 nm, respectively (Figure 7E,F). The unmodified His715 hence results in a slightly larger protein–protein distance and more fluctuating interactions with the ligand, albeit the radius of gyration and total interaction energies are essentially identical between the protein complexes.

#### 3.3.3 eEF2–ETA Complex with Non-bonded ADP-Ribose

**Figure 8:**
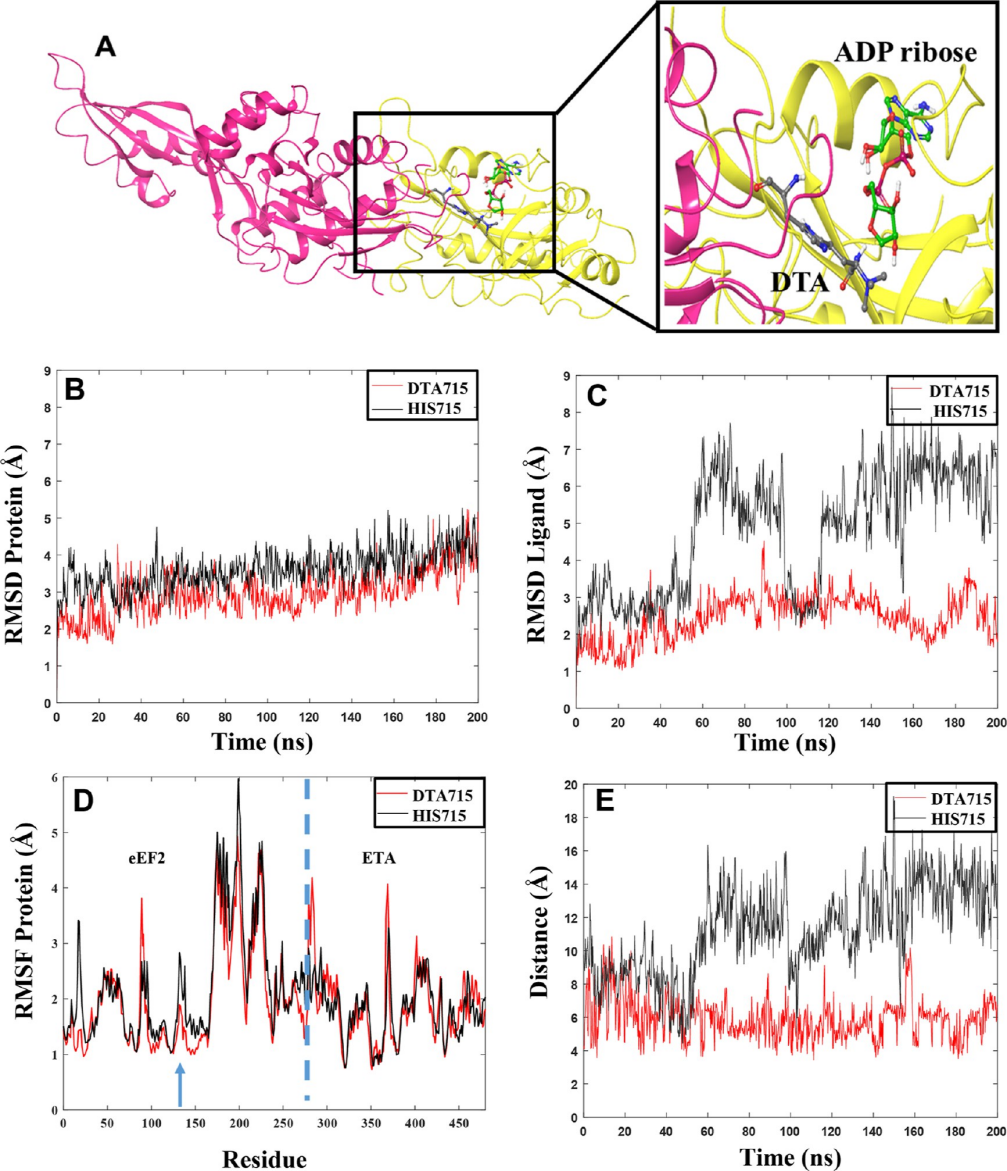
(A) Generated complex between ETA (yellow) containing ADP-ribose and human eEF2 (pink) with DTA in position 715. (B) Rmsd values for the protein complexes from one of the 200 ns MD replicas. (C) Rmsd values for the ADP-ribose ligand in the presence of DTA (red) or His715 (black) in eEF2; same replicas as in 8B. (D) RMSF values for individual residues. The dashed blue line separates protein in the complex. Residues 1–279 correspond to eEF2, and residues 280–486 correspond to ETA. The blue arrow corresponds to the location of DTA715 and His715. (E) Fluctuation of the distance between the reactive carbon atom in ADP-ribose and the N3 atom in DTA715 (red) or His715 (black) during same replica.

After release of the nicotinamide unit in NAD^+^, yielding the ETA-ADP-ribose–eEF2 complex, it is interesting to analyze if the reacting atoms in ADP-ribose and DTA715 (or His715) are in close proximity or not, which will provide further insight into the mechanism. To this end, the PDB structure containing the ETA-NAD^+^–*y*EF2 complex (PDB ID:2ZIT) was selected, and all chains except one copy of ETA were removed. To form ADP-ribose, the nicotinamide unit from NAD^+^ was removed, and the terminal carbon atom was positively charged. Finally, the protein was merged with the truncated homology model of *h*EF2 containing the modified residue DTA715 (Figure 8A). The complex was prepared in Maestro, minimized, and subjected to MD simulations as above.

Rmsd and RMSF values were calculated for the two complexes studied, *i.e*., for eEF2 having DTA or His in position 715 (Figure 8B–D, Table S4, and Figure S6).

**Figure 9:**
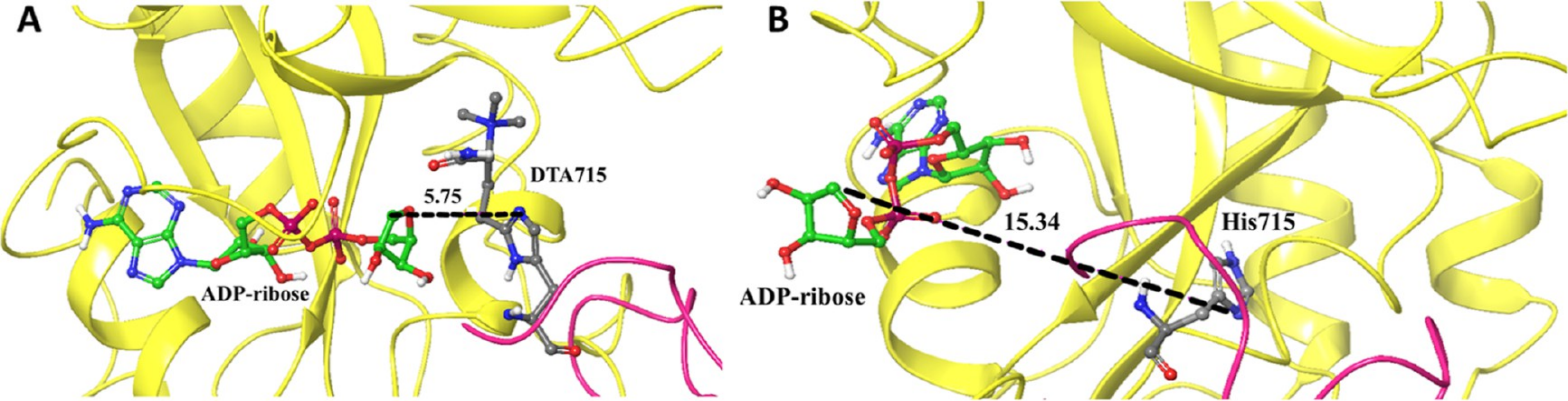
Representative snapshots of the eEF2–ETA complex with ADP-ribose as ligand during one of the MD simulation replicas. (A) Structure of the eEF2–ETA complex with ADP-ribose and DTA715, after 60 ns. (B) Structure of the eEF2–ETA complex with ADP-ribose and His715, after 60 ns. The ligand is displaced from the cavity and located far away from His715.

The rmsd values for both proteins show a slow but steady increase, indicating that the systems in these particular replicas are not completely stable (Figure 8B). One of the replicas for the system containing DTA715 (Figure S6 and Table S4) has an average rmsd for the protein of 4.48 Å, higher than the other two, although the total average over all three runs (3.45 Å) is lower than the same in the His715 containing system (4.02 Å). In case of the latter system, none of the three replicas seem to have stabilized, whereas in the case of the system with DTA715, it is only for that one replica shown in Figure 8B that the protein rmsd has not reached a steady state. Also, the ligand rmsds are larger for the His715 containing system (6.45 vs 4.49 Å, respectively; Table S4 and Figure S6). This is clearly illustrated in the rmsd plots in Figure 8C, from the same replicas as the plots in Figure 8B. For DTA715, the structural changes in the ligand are in all three replicas related to the ribose ring, previously bound to the nicotinamide unit (Figures 8C and S6). However, the ligand reaches a steady equilibrium in close proximity to the diphthamide unit (Figure 9A).

In the His715 complex there, is a sharp increase in the rmsd value for the ligand (Figure 8C). The large jump after 60 ns indicates that the ligand moves away from its initial binding site (Figure 9B) and loses the interactions with eEF2. Dissociation of the ligand from the protein is seen in all triplicates (Figure S6). The presence of His715 (absence of the diphthamide modification) thus appears to have an effect on the complex formation, and eEF2 with unmodified His will most likely not undergo ribosylation upon binding to ETA.

In the protein RMSF graph, analysis of the fluctuation score gives a higher value for His715 (1.98 Å) compared to DTA715 (1.61 Å) (Figure 8D, blue arrow). Considering both the RMSF and the rmsd values (Figure 8B), we conclude that unmodified His715 has a negative impact on the complex stability. It can also be inferred that DTA plays a key role in the formation of a productive complex with ADP-ribose and is properly oriented for the nucleophilic attack in the next step of the reaction. All data were confirmed in the triplicate replicas (Table S4 and Figure S6).

The distance plot for the system containing DTA715 (Figure 8E) shows only small changes during the trajectory and displays shorter distance values than that observed for the system containing His715 Figure 9. For the latter, large conformational changes are noted which correlates with the ligand rmsd (Figure 8C), indicating again that when His715 is present, the atoms aimed at forming the covalent bond are not properly positioned for the reaction to take place (Figure 9B). For DTA715, the ADP-ribose moiety is positioned at a proper distance for the next step in the ribosylation process.

The above data are further confirmed by the R_g_ analysis (Figure 7A,B), with larger conformational changes observed for the system containing His715 than for the DTA715 containing protein.

Comparing with the NAD^+^ bound systems, less negative interaction energy is noted (Figure 7C) for the ADP-ribose non-bonded system containing DTA715. Even so, the system has stronger interaction energy compared to the corresponding His715 containing system (Figure 7D).

The average values of the COM distances between the two proteins for the system containing DTA715 and His715 are 2.40 and 2.63 nm, respectively, meaning that the complex with unmodified His in position 715 is further apart (Figure 7E,F). This system furthermore displays large fluctuations, with COM distances frequently being above 3 nm.

#### 3.3.4 eEF2–ETA Complex with ADP Ribose Covalently Bound to DTA

As described in Figure 1, the last step in the eEF2 modification by ETA is the attachment of the ADP ribose moiety to the diphthamide imidazole ring, a modification that irreversibly inactivates the translation function of EF2 leading to cell death. To analyze the impact of ADP-ribose binding to the N3 atom of the diphthamide imidazole ring in eEF2 and the stability of the ADP-ribosylated eEF2–ETA complex, ADP-ribosylated yeast EF2 with DTA and ETA (PDB:1ZM2) was selected as starting point for the study.

**Figure 10:**
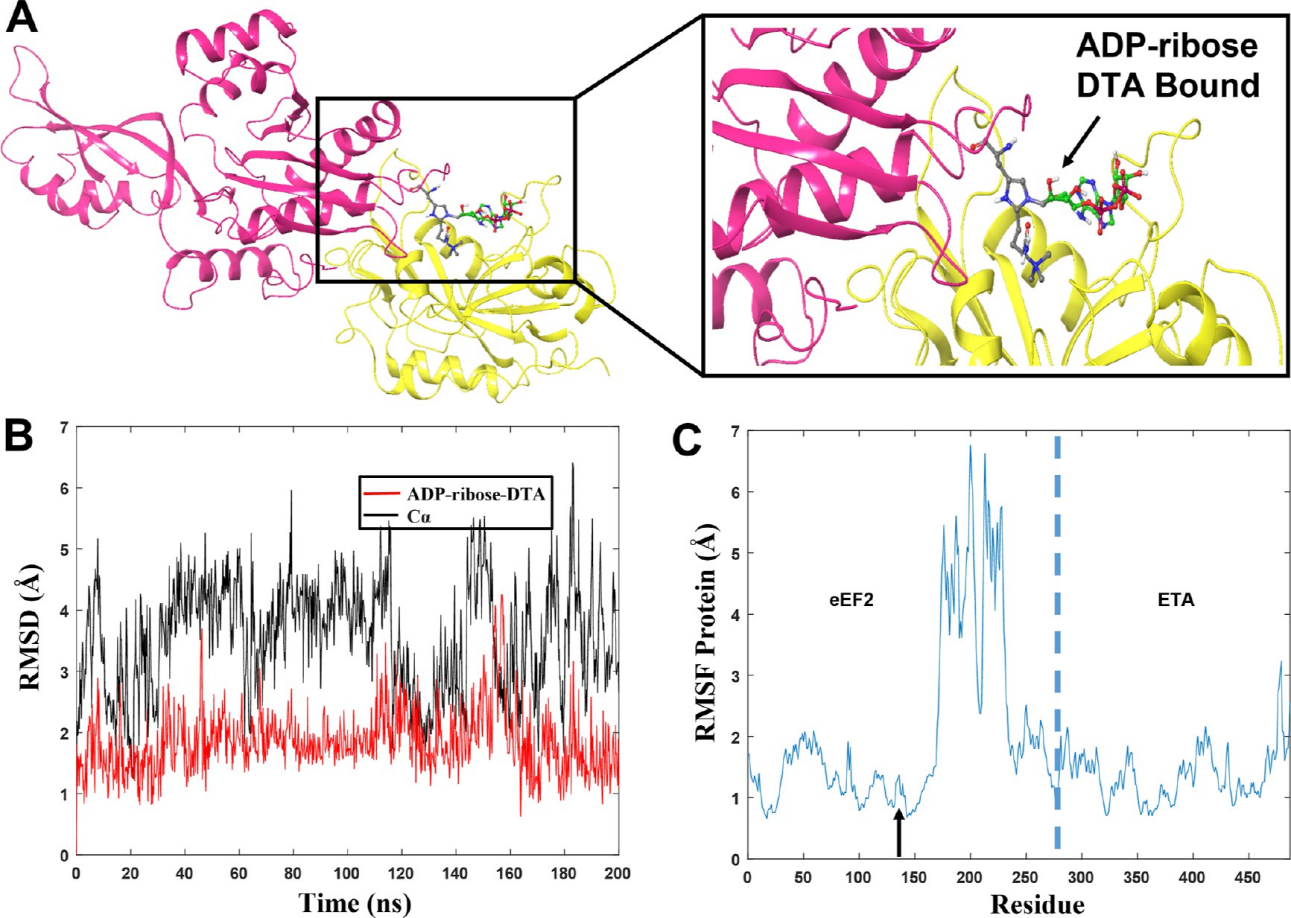
(A) Generated complex between ETA (yellow) and *h*EF2 (pink) containing ADP ribose bound to DTA715. The covalent bond is indicated by a black arrow. (B) Rmsd values for the protein Cα atoms and the ligand versus time for the ETA-eEF2 protein complex (black) and ADP-ribose-DTA ligand (red), which is covalently bound to eEF2, during one of the three 200 ns MD simulation replicas. (C) RMSF values for individual residues during the same replica. The dashed blue line separates the two chains of the protein complex. Residues 1–279 correspond to eEF2, and residues 280–486 correspond to the ETA. Location of DTA715 is indicated by the black arrow.

We then overlapped the structure of 1ZM2 with the homology model of human eEF2. After superposing the proteins (rmsd 1.94 Å), we removed the chain corresponding to the yeast eEF2, the proteins were merged, and a covalent bond was created between the carbon atom of the ADP-ribose moiety and the N3 atom of DTA (Figure 10A), followed by equilibration and 200 ns MD simulations in triplicate.

Figure 10B shows that the trajectory rmsd of the ligand (red curve) is relatively stable, with some fluctuations. These deviations are related to the adenine group of ADP-ribose that is slightly exposed to the solvent. When comparing to the protein rmsd for the NAD^+^ complex with eEF2–ETA (Figure 6B, red curve), higher fluctuations are observed in the protein complex in the ADP-ribose bound system (Figure 10B, black curve), which can be seen as an indication that the reaction affects the stability of the complex. We note that although the proteins do not dissociate from each other during any of the 200 ns MD simulations, the protein rmsds undergo large fluctuations, and each replica displays high (∼3.5 Å) average rmsd values (Table S5 and Figure S7). Longer simulation times may enable full dissociation of the protein complex to generate free ETA that can bind to a new molecule of NAD^+^ and start a new cycle of ribosylation.

According to the radius of gyration shown in Figure 7A, it is again clear that some larger fluctuations are observed for the covalently bound system compared to the system containing NAD^+^ as a ligand. Regarding the protein–protein interaction energies, ADP-ribose bound to DTA displays the weakest interactions in comparison with the other ligands (Figure 7C). This further supports that once the ribosylation has taken place, ETA may readily detach and bind a new NAD^+^ molecule as mentioned above. The average COM distance is 2.95 nm (Figure 7E), indicating a lower protein–protein complex stability in comparison with those having NAD^+^ or non-bonded ADP-ribose as ligands. All the above data were calculated only for the modified system (DTA715) with covalent ADP-ribose covalently bound, given that the simulations clearly indicated that His715 will not react with ADP-ribose.

#### 3.3.5 eEF2–ETA Complex with βTAD

**Figure 11:**
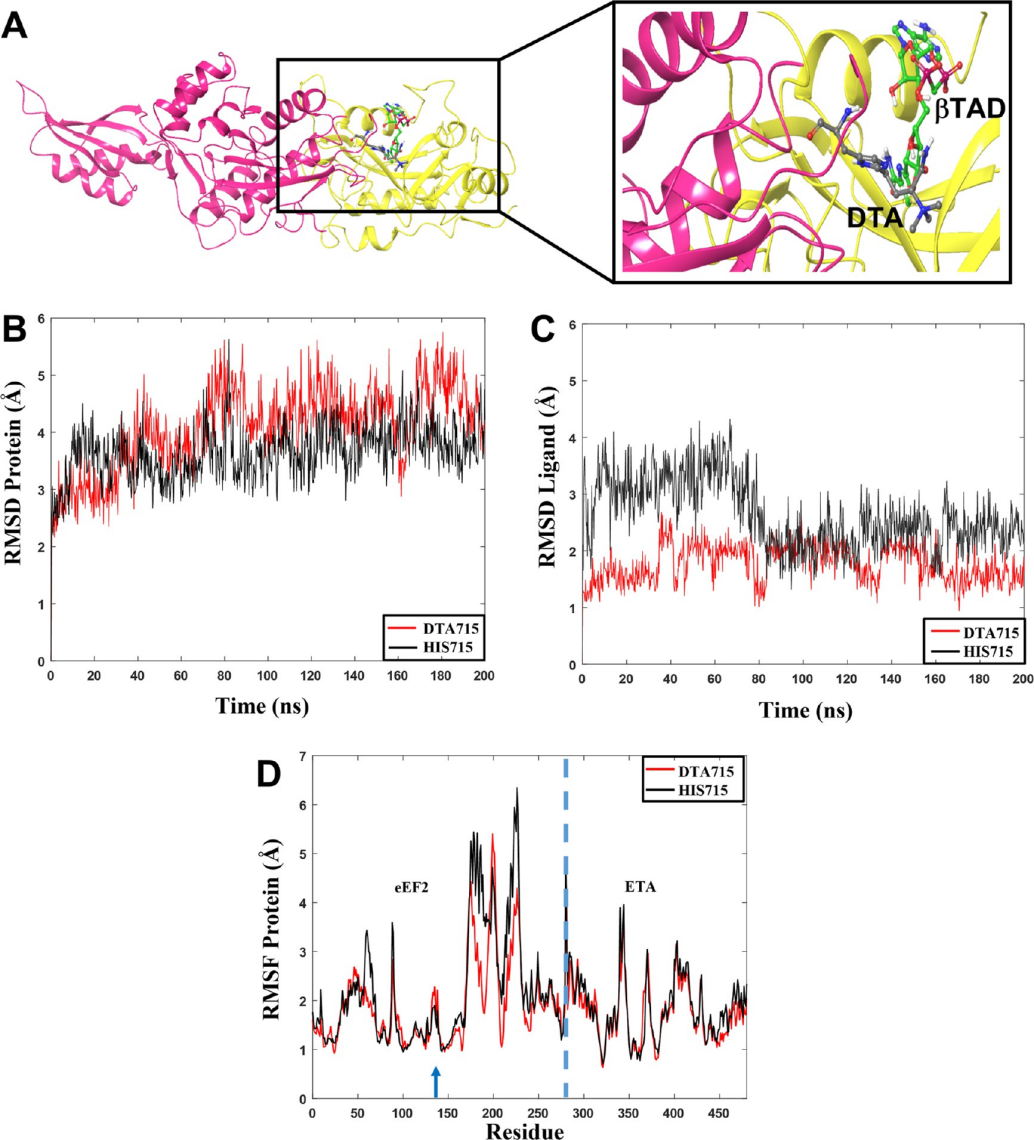
(A) Generated complex between ETA (yellow) containing βTAD and *h*EF2 (pink) with DTA in position 715. (B) Rmsd values for the protein complexes and (C) βTAD ligand in the presence of DTA (red) or His715 (black) in eEF2. (D) RMSF values for individual residues All data from one of the three 200 ns MD simulation replicas. The dashed blue line separates two chains of the protein complex. Residues 1–279 correspond to eEF2 and residues 280–486 correspond to ETA. The blue arrow corresponds to the location of DTA715 and His715.

Aiming to compare the EF2-ETA complexes containing the natural substrate (NAD^+^) with that containing an inhibitory analogue, we selected the PDB structure 1ZM4 with βTAD. DTA was thereafter introduced to explore how the modified residue affects the protein–protein interactions during the MD trajectory. The NAD^+^ analogue βTAD is placed in the same orientation within the active site and has similar interactions as NAD^+^, especially concerning some crucial amino acids such as His440 and Tyr481 (Figure 5B). The *y*EF2 in 1ZM4 was overlapped with the previously truncated homology model of *h*EF2 containing the modified residue DTA715. The resulting rmsd was 3.46 Å, and after removing the chain corresponding to the yeast eEF2, proteins were merged, and the complex ETA-βTAD-*h*EF2 (DTA) was generated (Figure 11A). The systems were prepared in Maestro, minimized, and subjected to MD simulations.

For the protein system containing DTA715 (Figure 11B, Table S6, and Figure S8), the rmsd values, though slightly higher than those observed for the His715 system, show very small changes that do not affect the structure of the complexes. The rmsd plots for the ligand (Figure 11C, Table S6, and Figure S8) of the His715 containing system show more fluctuations and larger average rmsds than that of DTA715. These observations are supported by analyzing the radius of gyration for both systems (Figure 7A,B). There are no significant fluctuations for the ETA-βTAD-EF2 system with respect to compactness during the simulation time when DTA is present, whereas the protein complex fluctuates considerably when containing His715. Negative protein–protein interaction energies are observed for both systems, with minor changes for the His715 system (Figure 7C,D).

The RMSF values for DTA715 and His715 show the same trends, that is, that the fluctuations are somewhat larger in the His715 containing system, in particular for the ETA part (Figure 11D, Table S6, and Figure S8).

The average COM distance values are 2.65 nm and 3.35 nm for the systems containing DTA and His (Figure 7E,F), respectively. The distance between the proteins has increased compared to the same systems containing NAD^+^. The larger distances along with the ∼10 kcal/mol reduced interaction energy in the βTAD containing complexes compared to the NAD^+^ containing ones, also indicate less stable structures.

## 4 Conclusions and Perspective

In this work, we report a large number of MD simulations and *in silico* studies including the post-translational modified DTA residue, aiming to shed light on the role of DTA in the ADP-ribosylation reaction of eEF2. ADP-ribosylation by *P. aeruginosa* ETA is only possible when the modified residue diphthamide is present in eEF2.^17,23^

The current study sheds light on several aspects of the ribosylation process. First, by analyzing the complexes between ETA and NAD^+^ or ADP-ribose, we conclude that the fluctuations in the latter case suggest that it is the ETA-NAD^+^ complex that binds to eEF2, and only then the nicotinamide moiety is released forming the complex ETA-eEF2-ADP-ribose. In addition, the results highlight the roles of Glu553 and Tyr481 in ETA for the nucleophilic attack, and a proper distance between the modified diphthamide and the ribose moiety of NAD^+^ was observed. Once the nicotinamide residue is released, the system is ready for the ribosylation reaction. When comparing the systems containing DTA715 or His715, we observed that in the latter, the ADP-ribose ligand moves away of the original binding site and will not reside in a proper position for the ribosylation reaction. These results confirm that eEF2 containing His715 is not likely to undergo ribosylation upon binding to ETA. The results for the system containing ADP-ribose covalently bound to DTA revealed that ribosylation affects the stability of the complexes. This suggests that right after ribosylation, ETA detaches and is ready to bind a new NAD^+^ molecule and thus able to ribosylate another eEF2 unit. Moreover, we analyzed the protein–protein–ligand complex structures by calculating radius of gyration, interaction energies, and COM distances. In all calculations, NAD^+^ as a ligand generated the most stable protein complex, and eEF2 with DTA715 throughout generates more stable systems than when the unmodified His715 is present. As expected, the inhibitor βTAD, an analogue of NAD^+^, leads to more stable complexes than when ADP-ribose, either bonded or non-bonded, is present as a ligand.

Having a better insight into the mechanism of ADP-ribosylation in the eEF2–ETA complex could serve as a starting point for the exploration of novel inhibitors against bacterial toxins by docking, MD simulations, and BPMD simulations to identify compounds binding better than NAD^+^ and thereby inhibit the deleterious ribosylation reaction of eukaryotic elongation factor 2.

## Data Availability

Data sets for the three replicas of 200 ns MD simulations of ETA with eEF2 and three different ligands: NAD^+^, ADP-ribose, and βTAD in the two systems (DTA715, His715); MD simulation trajectories of ETA with the different ligands; and metadynamics simulations of ETA with NAD^+^, ADP ribose, and βTAD are available for free download from the Zenodo archive: doi.org/10.5281/zenodo.7492351.

## Supporting Information Available

Data of homology modeling; BPMD simulations of NAD+, ADP-ribose, and βTAD bound to ETA; superposed structure of the ETA-NAD^+^–yeast eEF2 complex with the homology model of human eEF2; comparative MD simulation data for EF2–ETA−βTAD complexes; and rmsd and RMSF values for all replicas of the eEF2–ETA complexes with NAD+, ADP-ribose bonded/non-bonded, and βTAD (PDF)
