# Supplementary material for: Structural Insights into Pseudomonas aeruginosa Exotoxin A–Elongation Factor 2 Interactions: A Molecular Dynamics Study
Source: J Chem Inf Model. 2023 Feb 20;63(5):1578–91. doi: 10.1021/acs.jcim.3c00064 (PMC10015456; doi:10.1021/acs.jcim.3c00064)
Supplement: Supplementary file 1 — ci3c00064_si_001.pdf [file ci3c00064_si_001.pdf]

SUPPORTING INFORMATION

Structural insights into *Pseudomonas aeruginosa* Exotoxin A – Elongation Factor 2 interaction: A Molecular Dynamics Study.

Asma Gholami[1,2], Dariush Minai-Tehrani[2], Sayyed Jalil Mahdizadeh[1], Patricia Saenz-Mendez[3], Leif A. Eriksson[1]*

[1]Department of Chemistry and Molecular Biology, University of Gothenburg, 405 30 Göteborg, Sweden

[2]Faculty of Life Sciences and Biotechnology, Shahid Beheshti University, Tehran, Iran

[3]Department of Engineering and Chemical Sciences, Karlstad University, 651 88, Karlstad, Sweden

*Corresponding author: leif.eriksson@chem.gu.se

```
        10         20         30         40         50
MVNFTVDQIR AIMDKKANIR NMSVIAHVDH GKSTLTDSLV CKAGIIASAR
        60         70         80         90        100
AGETRFTDTR KDEQERCITI KSTAISLFYE LSENDLNFIK QSKDGAGFLI
       110        120        130        140        150
NLIDSPGHVD FSSEVTAALR VTDGALVVVD CVSGVCVQTE TVLRQAIAER
       160        170        180        190        200
IKPVLMMNKM DRALLELQLE PEELYQTFQR IVENVNVIIS TYGEGESGPM
       210        220        230        240        250
GNIMIDPVLG TVGFGSGLHG WAFTLKQFAE MYVAKFAAKG EGQLGPAERA
       260        270        280        290        300
KKVEDMMKKL WGDRYFDPAN GKFSKSATSP EGKKLPRTFC QLILDPIFKV
       310        320        330        340        350
FDAIMNFKKE ETAKLIEKLD IKLDSEDKDK EGKPLLKAVM RRWLPAGDAL
       360        370        380        390        400
LQMITIHLPS PVTAQKYRCE LLYEGPPDDE AAMGIKSCDP KGPLMMYISK
       410        420        430        440        450
MVPTSDKGRF YAFGRVFSGL VSTGLKVRIM GPNYTPGKKE DLYLKPIQRT
       460        470        480        490        500
ILMMGRYVEP IEDVPCGNIV GLVGVDQFLV KTGTITTFEH AHNMRVMKFS
       510        520        530        540        550
VSPVVRVAVE AKNPADLPKL VEGLKRLAKS DPMVQCIIEE SGEHIIAGAG
       560        570        580        590        600
ELHLEICLKD LEEDHACIPI KKSDPVVSYR ETVSEESNVL CLSKSPNKHN
       610        620        630        640        650
RLYMKARPFP DGLAEDIDKG EVSARQELKQ RARYLAEKYE WDVAEARKIW
       660        670        680        690        700
CFGPDGTGPN ILTDITKGVQ YLNEIKDSVV AGFQWATKEG ALCEENMRGV
       710        720        730        740        750
RFDVHDVTLH ADAIHRGGGQ IIPTARRCLY ASVLTAQPRL MEPIYLVEIQ
       760        770        780        790        800
CPEQVVGGIY GVLNRKRGHV FEESQVAGTP MFVVKAYLPV NESFGFTADL
       810        820        830        840        850
RSNTGGQAFP QCVFDHWQIL PGDPFDNSSR PSQVVAETRK RKGLKEGIPA

                       LDNFLDKL
```

Figure S1. Human eEF2 amino acid sequence

Table S1. Parameters for the homology modeling

**Modeling speed (slow = best)**	**slow**
Number of PSI-BLAST iterations in template search	6
Maximum allowed PSI-BLAST E-value to consider template	0.5
Maximum number of templates to be used	5
Maximum number of templates with the same sequence	1
Maximum oligomerization state	4
Maximum number of alignment variations per template	5
Maximum number of conformations tried per loop	50
Maximum number of residues added to the termini	10

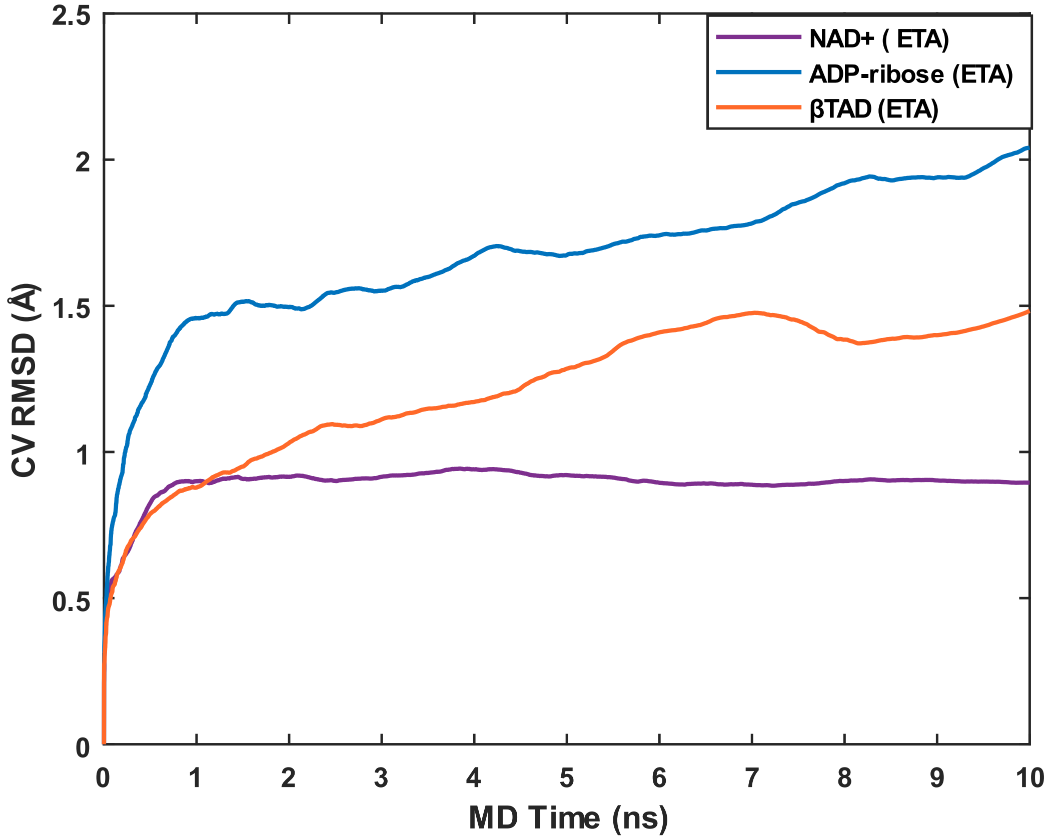

Figure S2. RMSD estimate averaged over 10 BPMD simulations vs simulation time for NAD^+, ADP-ribose and βTAD bound to ETA.

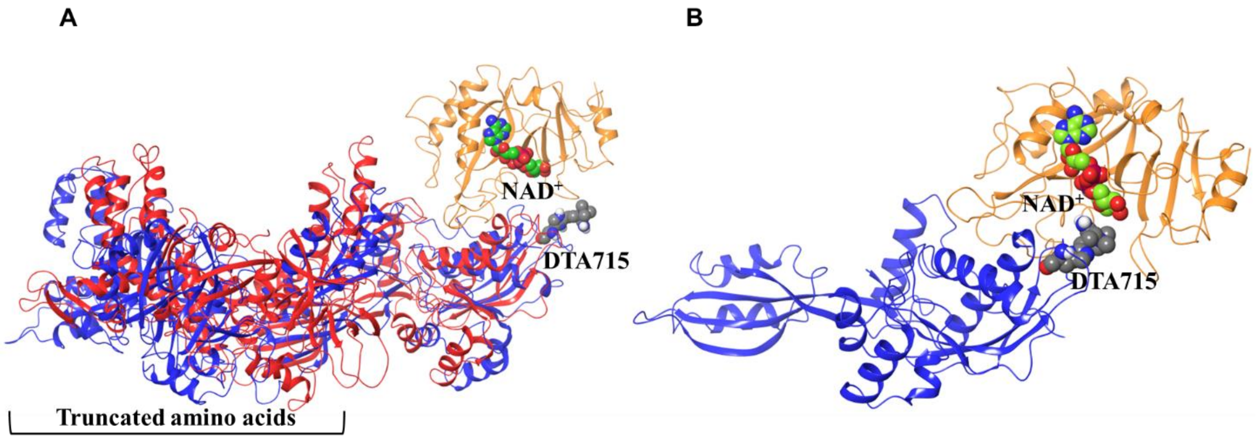

Figure S3. (A) The overlay structure of ETA-NAD^+ - yeast eEF2 complex (PDB ID: 2ZIT) (ETA with NAD^+ as a ligand in orange and *y*EF2 in red) with the homology model of human eEF2 (blue) containing DTA715. (B) Model of human EF2 after truncation of residues 1-578, merged with ETA.

Table S2. Average RMSD values (3 replica) for ETA and with 3 different ligands during 200 ns MD simulations.

	Average RMSD of Protein (Å)			Average RMSD of Ligand (Å)		
	**NAD$^+$**	**ADP-ribose**	**βTAD**	**NAD$^+$**	**ADP-ribose**	**βTAD**
**Replica 1**	1.90	2.20	1.98	1.89	6.84	1.84
**Replica 2**	1.70	1.58	1.89	1.60	5.15	1.63
**Replica 3**	1.94	1.97	2.19	1.42	3.68	1.77

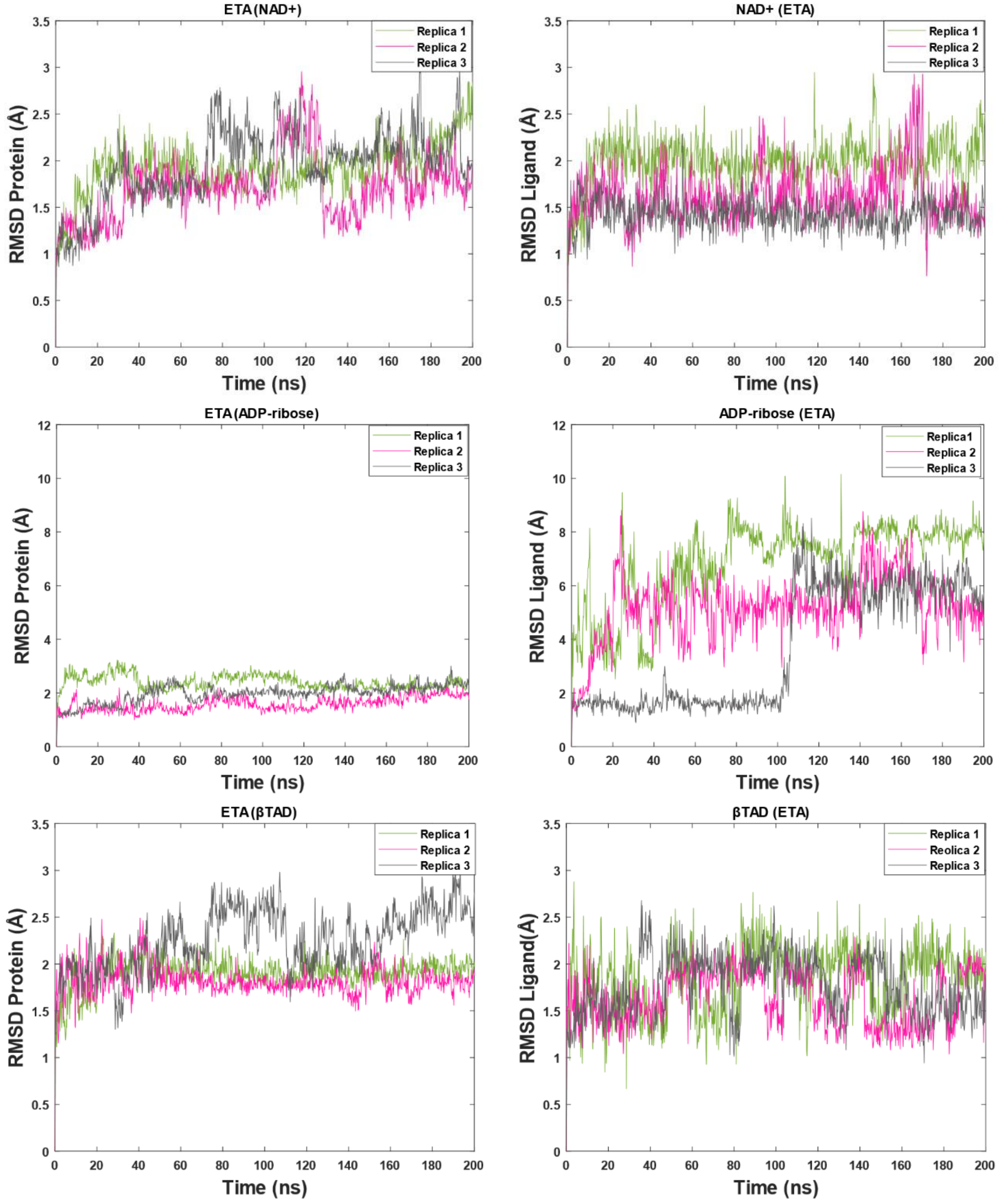

Figure S4. RMSD values (200 ns MD simulations in 3 replica) for ETA and the three ligands NAD$^+$, ADP-ribose and βTAD.

Table S3. Average RMSD and RMSF values (3 replica) for the eEF2 – ETA complex with NAD^{+} during 200 ns MD simulations.

	Average RMSD of Protein (Å)		Average RMSD of Ligand (Å)		Average RMSF of Protein (Å)	
	**DTA715**	**HIS715**	**DTA715**	**HIS715**	**DTA715**	**HIS715**
**Replica 1**	4.09	3.97	2.03	2.21	1.74	1.94
**Replica 2**	3.24	3.32	1.94	2.09	1.57	1.96
**Replica 3**	2.83	3.66	1.67	1.89	1.80	1.82

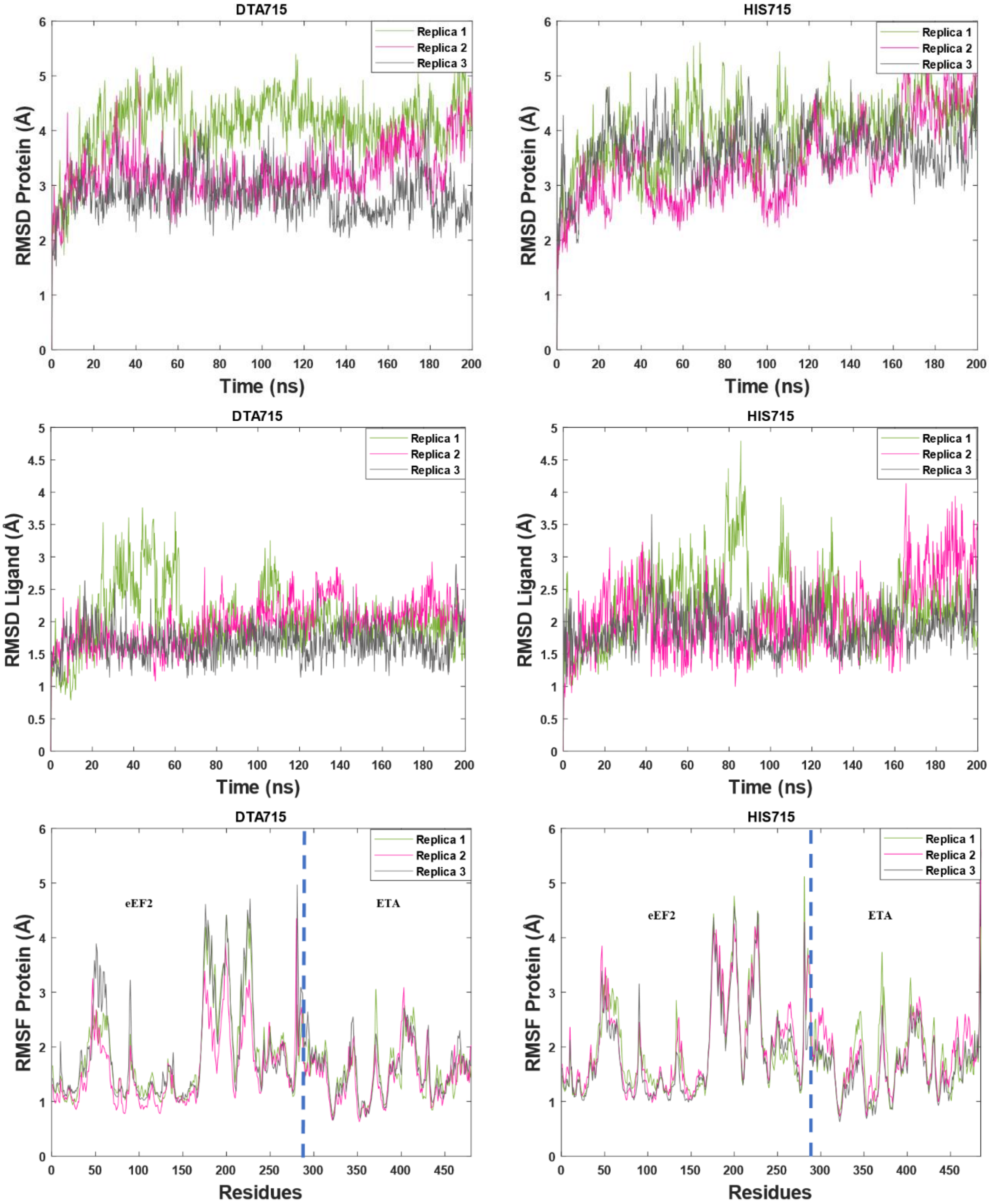

Figure S5. RMSD and RMSF values (200 ns MD simulations in 3 replica) for the eEF2 – ETA complex with NAD^{+}.

Table S4. Average of RMSD, and RMSF values (3 replica) for the eEF2 – ETA complex with non-bonded ADP-ribose during 200 ns MD simulations.

	**Average RMSD of Protein (Å)**		**Average RMSD of Ligand (Å)**		**Average RMSF of Protein (Å)**	
	**DTA715**	**HIS715**	**DTA715**	**HIS715**	**DTA715**	**HIS715**
**Replica 1**	4.48	4.30	5.68	9.72	2.20	2.21
**Replica 2**	2.92	4.17	5.34	4.82	1.64	2.40
**Replica 3**	2.96	3.60	2.45	4.80	1.94	2.07

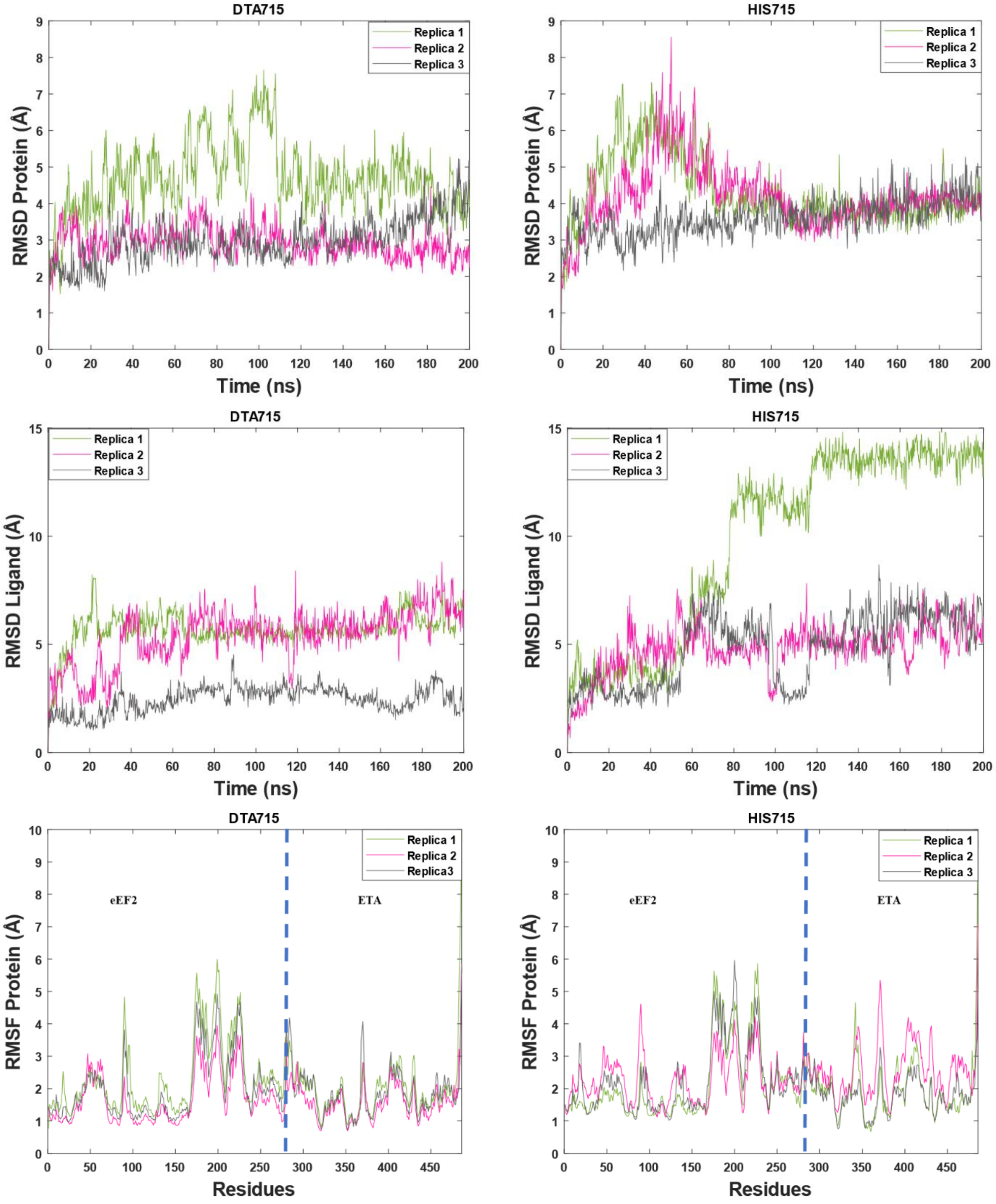

Figure S6. RMSD and RMSF values (200 ns MD simulations in 3 replica) for the eEF2 – ETA complex with non-bonded ADP-ribose.

Table S5. Average of RMSD and RMSF values (3 replica) for the eEF2 – ETA complex with ADP ribose covalently bound to DTA during 200 ns MD simulations.

	**Average RMSD of Protein (Å)**	**Average RMSD of Ligand (Å)**	**Average RMSF of Protein (Å)**
	**DTA715**	**DTA715**	**DTA715**
**Replica 1**	3.65	1.20	1.83
**Replica 2**	3.38	1.40	1.30
**Replica 3**	3.56	1.84	1.77

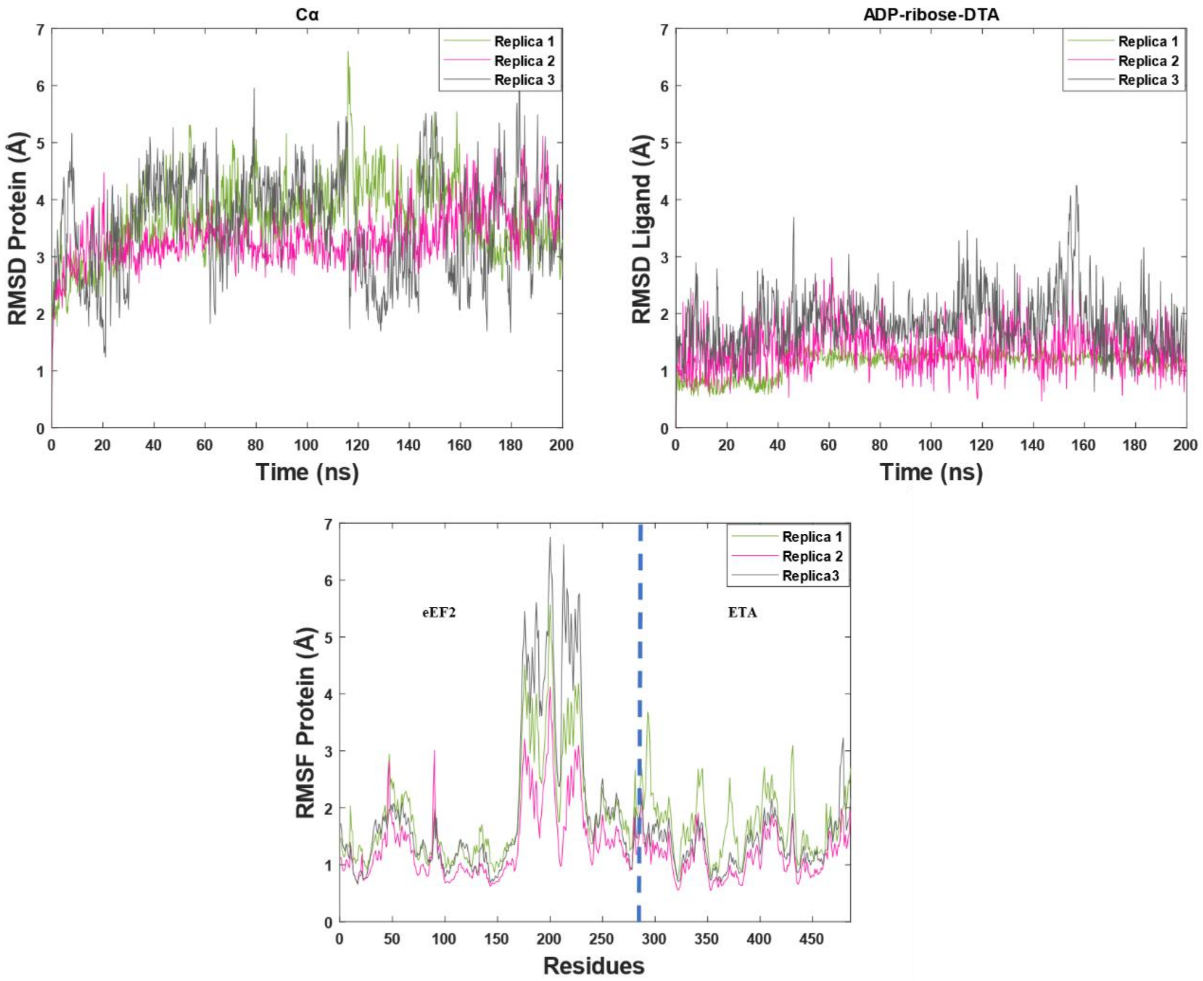

Figure S7. RMSD and RMSF values (200 ns MD simulations in 3 replica) for the eEF2 – ETA complex with ADP ribose covalently bound to DTA.

Table S6. Average of RMSD and RMSF values (200 ns MD simulations in 3 replica) for the eEF2 – ETA complex with βTAD.

	Average RMSD of Protein (Å)		Average RMSD of Ligand (Å)		Average RMSF of Protein (Å)	
	DTA715	HIS715	DTA715	HIS715	DTA715	HIS715
**Replica 1**	4.03	3.93	2.57	3.93	1.90	2.71
**Replica 2**	3.89	3.53	2.61	2.63	2.10	2.11
**Replica 3**	4.07	3.65	2.01	2.58	1.87	2.05

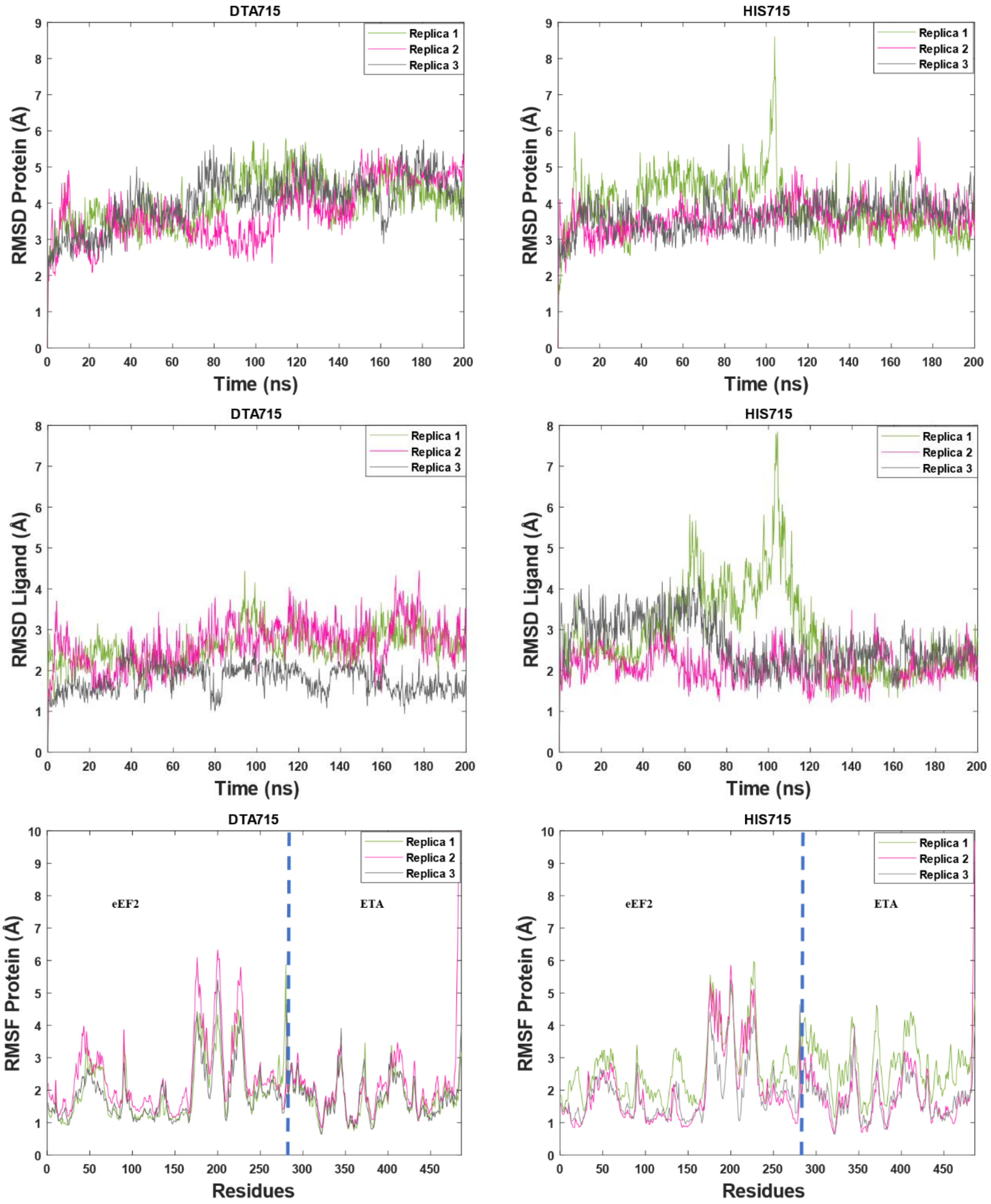

Figure S8. RMSD and RMSF values (200 ns MD simulations in 3 replica) for the eEF2 – ETA complex with βTAD.
